# Cost-Effectiveness of Valve-in-Valve Transcatheter Mitral Valve Replacement Versus Redo Surgical Mitral Valve Replacement for Degenerated Bioprosthetic Mitral Valve

**DOI:** 10.1016/j.shj.2026.100808

**Published:** 2026-02-09

**Authors:** Elina E. Pliakos, Kriyana Reddy, Pavan Atluri, Howard C. Herrmann, Paul Fiorilli, Mohamad Alkhouli, Suzanne J. Baron, Jay Giri, Ashwin S. Nathan

**Affiliations:** a Division of Cardiovascular Medicine, University of Pennsylvania Perelman School of Medicine, Philadelphia, Pennsylvania, USA; b Penn Cardiovascular Outcomes, Quality, and Evaluative Research Center, University of Pennsylvania, Philadelphia, Pennsylvania, USA; c Division of Cardiovascular Surgery, University of Pennsylvania Perelman School of Medicine, Philadelphia, Pennsylvania, USA; d Division of Cardiology, Mayo Clinic, Rochester, Minnesota, USA; e Division of Cardiology, Massachusetts General Hospital, Boston, Massachusetts, USA; f Baim Institute for Clinical Research, Boston, Massachusetts, USA

**Keywords:** Bioprosthetic valve degeneration, Cost-effectiveness analysis, Decision-analytic model, Mitral valve-in-valve, Redo surgical mitral valve replacement, Transcatheter mitral valve replacement

## Abstract

**Background:**

Degenerated bioprosthetic mitral valves (MVs) are associated with significant morbidity and health care expenditures. For certain patients, valve-in-valve transcatheter MV replacement (ViV TMVR) has emerged as a promising treatment option due to fewer complications and shorter hospital length of stay when compared with redo surgical MV replacement (redo-SMVR). We constructed a decision-analytic model comparing the cost-effectiveness of ViV TMVR to redo-SMVR for the management of degenerated bioprosthetic valves.

**Methods:**

Cost-effectiveness was determined by calculating deaths averted and incremental cost-effectiveness ratios (ICERs). Uncertainty was addressed by plotting cost-effectiveness planes and acceptability curves for various willingness-to-pay thresholds. The main outcome was ICERs defined as United States (US) dollars/deaths averted.

**Results:**

In the base case analysis, the cost associated with ViV TMVR was estimated at $87,724 with a 0.93 probability of survival at 1 month. For the redo-SMVR strategy, the cost was $104,444, and the probability of survival at 1 month was 0.89. Overall, ViV TMVR resulted in savings of $418,001 per death averted (ICER, −$418,001/death averted). In cost-effectiveness acceptability curves, ViV TMVR was cost-effective in 83% to 88% of simulations for a willingness-to-pay threshold ranging from $0 to $100,000.

**Conclusions:**

ViV TMVR is an effective strategy that may result in significant health care savings for the management of degenerated bioprosthetic valves.

## Highlights

• Decision model compared valve-in-valve transcatheter mitral valve replacement (ViV TMVR) vs. redo-surgical mitral valve replacement for failed bioprosthetic mitral valve.
• At 1 month, ViV TMVR was less costly and more effective (dominant).
• ViV TMVR was cost-effective in 83%–88% of simulations across willingness-to-pay $0–$100k.
• Cost-effectiveness was driven mainly by length of stay and valve/device costs.

## Graphical abstract

**Figure:**
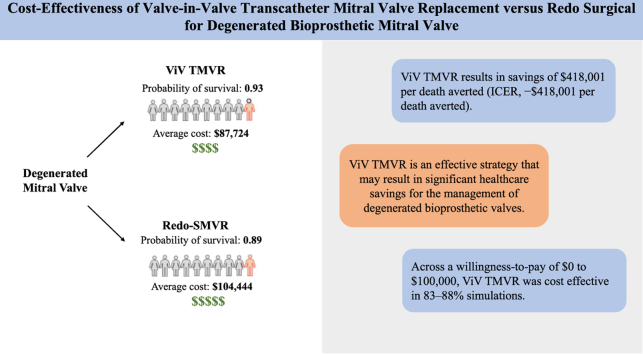

## Introduction

Degenerated bioprosthetic mitral valves (MVs) are associated with significant morbidity and health care expenditures.^1^ The 15-year survival rate after bioprosthetic MV replacement has been reported at 40%,^2^ and 10-year actuarial freedom from structural valve deterioration at 70.2%.^3^ Presently, the management of degenerated bioprosthetic MVs presents a choice between surgical and percutaneous options, with redo surgical MV replacement (redo-SMVR) serving as the established gold standard. Treatment selection, however, is also influenced by patient surgical risk, comorbidity burden, anatomical suitability, and valve-specific characteristics. Valve-in-valve transcatheter MV replacement (ViV TMVR) has emerged as a promising alternative that is associated with fewer complications and shorter hospital length of stay (LOS).^4^ This less invasive approach involves the insertion of a new transcatheter valve within the failed bioprosthetic valve. Notably, both replacement methods are resourceintensive and costly, imposing a burden on the health care system and patients. As the use of bioprosthetic MVs has dramatically risen from an estimated 16.8% in 1996 to 53.7% in 2013,^5^ the number of patients presenting with failing bioprosthetic valves is anticipated to increase in the near future.^6^ This anticipated growth in demand for reintervention underscores the importance of identifying management strategies that not only achieve favorable clinical outcomes but also represent the efficient use of limited health care resources. In light of the need to pinpoint high-value strategies that enhance patient care while managing expenditures, the aim of this study was to perform a cost-effectiveness analysis comparing ViV TMVR to redo-SMVR for the management of degenerated bioprosthetic MVs.

## Methods

### Model Structure

**Figure 1:**
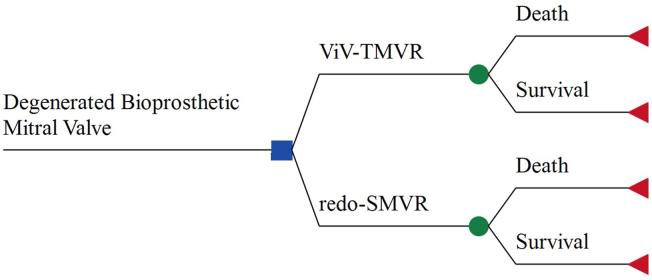
**Decision tree model.** The square indicates the decision to choose between the use of ViV TMVR or redo-SMVR for the management of degenerated bioprosthetic mitral valves. The circles indicate chance nodes, and the triangles, endpoints. Abbreviations: redo-SMVR, redo surgical mitral valve replacement; ViV TMVR, valve-in-valve transcatheter mitral valve replacement.

We constructed a decision analytic model (Figure 1) assessing the cost-effectiveness of ViV TMVR compared to redo-SMVR for the management of degenerated bioprosthetic MVs. The patient population of our analysis consisted of adult hospital in-patients undergoing ViV TMVR or redo-SMVR for a degenerated bioprosthetic MV. Costs and outcomes were calculated for a time horizon of 1 month, and the analysis was performed from a societal perspective. Capturing the full economic impact of health interventions on society, the societal perspective is a viewpoint that incorporates all costs and health effects of interventions regardless of who incurs the costs or who obtains the effects.

Our study included an impact inventory, as recommended by guidelines.^7^ The impact inventory is a checklist of health and nonhealth outcomes that were considered in this analysis, and it can be found in Table 1. Cost data were obtained from sources that reported values in United States (US) dollars. Mortality was defined as 30-day mortality. The model was developed using the software TreeAge Pro 2019 (TreeAge, Williamstown, Massachusetts).

**Table 1:** Impact inventory

Sector	Type of impact	Classification	Included in this analysis from the societal perspective?
Formal health care sector			
Health	Health outcomes (effects)		
	Mortality		✔
	*Medical costs*		
	Paid for by third-party payers	Direct	✔
	Paid for by patients out-of-pocket	Direct	✔
	Future related medical costs (payers and patients)	Direct	✔
	Future unrelated medical costs (payers and patients)		✘
Nonhealth care sector			
Productivity	Labor market earnings lost due to absence from work	Indirect	✔
	Uncompensated household production, patient	Indirect	✘

Direct costs refer to medical expenditures related to health care utilization, whereas indirect costs reflect productivity losses outside the formal health care sector.

The analysis followed the recommendations made by the Consolidated Health Economic Evaluation Reporting Standards Statement^8^ and the guidelines reported in 2017 by the Second Panel on Cost-Effectiveness Analysis.^7^ This study did not require institutional review board approval, as it used data from the publicly available literature.

### Model Inputs: Assigning Probabilities

Our decision model had two treatment arms. To identify studies that provide data on the effectiveness of each treatment arm (namely ViV TMVR vs. redo-SMVR) and that directly compare them, we used the most relevant systematic reviews and meta-analyses.^4^^,^^9^, ^10^, ^11^, ^12^, ^13^, ^14^ After screening the literature and the review studies, we only included retrospective studies that provided a direct comparison between the two strategies.^15^, ^16^, ^17^, ^18^, ^19^, ^20^ Studies that used data from the National Inpatient Sample^21^^,^^22^ and the National Readmission Database^23^ were excluded to avoid duplicate counts. Probability estimates and CIs for mortality, risk of stroke, bleeding, acute kidney injury, arrhythmia, and pacemaker placement were obtained by pooling with the use of random-effects meta-analysis (Der Simonian and Laird) (MedCalc V19.8)^24^ for each treatment arm separately. This method was chosen as it accounts for the considerable interstudy differences and heterogeneity among the included studies.^24^ To estimate a different LOS for each treatment strategy, we calculated a weighted average of LOS based on four studies that provided data for both strategies.^15^, ^16^, ^17^^,^^20^

### Model Inputs: Assigning Costs

Costs were obtained from the literature and adjusted to January 2024 US dollars using the consumer price index inflation calculator provided by the Bureau of Labor Statistics.^25^

The cost estimate for SMVR was obtained from a study by Atluri *et al.,*^26^ and it included the costs of operative supplies, operative time, perfusion, nursing, laboratory, blood bank, and pharmacy. The procedural cost for ViV TMVR was estimated from a study by Baron *et al*.,^27^ which accounted for the costs of room/depreciation, nonphysician personnel, and physician fees. For the cost of the valve itself, we assumed a base case cost of $39,654 (adjusted) based on the reported costs of an Edwards Sapien 3 Transcatheter Valve ($32,500 unadjusted).^28^ The costs of complications, including the cost of a permanent pacemaker, arrhythmia, renal insufficiency, major stroke, and bleeding, were obtained from a study by Arnold *et al*.^29^ that calculated costs of periprocedural complications for patients treated with transcatheter aortic valve replacement.

The cost of hospitalization was estimated by multiplying the cost of hospitalization per day for the state of Pennsylvania, provided by the Kaiser Family Foundation,^30^ with the length of hospital stay for patients who received ViV TMVR or redo-SMVR.^15^, ^16^, ^17^^,^^20^ The cost of lost productivity per day was estimated by multiplying the usual daily earnings for US salary workers provided by the US Department of Labor^25^ by the LOS associated with ViV TMVR or redo-SMVR. Postprocedural follow-up costs, including rehab/Skilled Nursing Facility stays, outpatient services, and physician fees, were obtained from the PARTNER 3 trial comparing transcatheter aortic valve replacement and surgical aortic valve replacement costs in the 0–30-day follow-up period.^31^

### Outcome and Data Analysis

In the base case analysis, our primary outcome was the incremental cost-effectiveness ratio (ICER), defined as the ratio of the incremental cost between the two strategies (ViV TMVR or redo-SMVR) over their incremental difference in effectiveness.^7^ The incremental cost was defined as the excess cost of ViV TMVR compared to the cost of redo-SMVR. In turn, the incremental effectiveness was defined in terms of deaths averted. Moreover, net monetary benefit was defined as the cost of the strategy subtracted from the effectiveness multiplied by the willingness-to-pay (WTP) threshold.

The robustness of our model was evaluated with the use of deterministic (one-way sensitivity) and probabilistic sensitivity analysis (Monte Carlo). In the one-way sensitivity analysis, each parameter was tested across a range of multiple point estimates, whereas in the probabilistic analysis, we varied all parameters of the model simultaneously. The deterministic (one-way) sensitivity analyses allowed us to evaluate the impact of individual parameters on model outcomes and identify key drivers of cost-effectiveness, while the probabilistic sensitivity analysis was used to quantify overall parameter uncertainty and assess the likelihood that ViV TMVR remains cost effective across a range of WTP thresholds. The base-case estimates, ranges, and distributions for all parameters are presented in Table 2.

**Table 2:** Model inputs and baseline estimates for probabilities, length of stay, and costs

	Base case value (range and distribution)	Source
Probabilities		
Probability of mortality with ViV TMVR	0.07 (range: 0.03-0.13) Beta (0.07; SD: 0.01)	Kamioka et al.,^15^ Simonettoet et al.,^16^ Zubarevich et al.,^17^ Simard et al.,^18^ Szlapka et al.^19^
Probability of mortality with redo-SMVR	0.11 (range: 0.07-0.16) Beta (0.11; SD: 0.02)	Kamioka et al.,^15^ Simonettoet et al.,^16^ Zubarevich et al.,^17^ Simard et al.,^18^ Szlapka et al.^19^
Probability of pacemaker placement with ViV TMVR	0.02 (range: 0-0.08) Beta (0.02; SD: 0.01)	Zubarevich et al.,^17^ Szlapka et al.^19^
Probability of pacemaker placement with redo-SMVR	0.15 (range: 0.09-0.22) Beta (0.15; SD: 0.02)	Zubarevich et al.,^17^ Szlapka et al.^19^
Probability of arrhythmia with ViV TMVR	0.08 (range: 0-0.22) Beta (0.08; SD: 0.04)	Kamioka et al.,^15^ Simonettoet et al.,^16^ Zubarevich et al.,^17^ and Szlapka et al.^19^
Probability of arrhythmia with redo-SMVR	0.33 (range: 0.25-0.41) Beta (0.33; SD: 0.03)	Kamioka et al.,^15^ Simonettoet et al.,^16^ Zubarevich et al.,^17^ Szlapka et al.^19^
Probability of bleeding with ViV TMVR	0.07 (range: 0.01-0.17) Beta (0.07; SD: 0.03)	Kamioka et al.,^15^ Simonettoet et al.,^16^ Zubarevich et al.,^17^ Szlapka et al.,^19^ Murzi et al.^20^
Probability of bleeding with redo-SMVR	0.28 (range: 0.14-0.44) Beta (0.28; SD: 0.05)	Kamioka et al.,^15^ Simonettoet et al.,^16^ Zubarevich et al.,^17^ Szlapka et al.,^19^ Murzi et al.^20^
Probability of acute kidney injury with ViV TMVR	0.06 (range: 0.01-0.16) Beta (0.06; SD: 0.03)	Simonettoet et al.,^16^ Zubarevich et al.,^17^ Szlapka et al.,^19^ Murzi et al.^20^
Probability of acute kidney injury with redo-SMVR	0.16 (range: 0.09-0.25) Beta (0.16; SD: 0.03)	Simonettoet et al.,^16^ Zubarevich et al.,^17^ Szlapka et al.,^19^ Murzi et al.^20^
Probability of stroke with ViV TMVR	0.01 (range: 0-0.03) Beta (0.01; SD: 0)	Kamioka et al.,^15^ Simonettoet et al.,^16^ Zubarevich et al.,^17^ Simard et al.,^18^ Szlapka et al.,^19^ Murzi et al.^20^
Probability of stroke with redo-SMVR	0.05 (range: 0.03-0.08) Beta (0.05; SD: 0)	Kamioka et al.,^15^ Simonettoet et al.,^16^ Zubarevich et al.,^17^ Simard et al.,^18^ Szlapka et al.,^19^ Murzi et al.^20^
Costs (US dollars)		
Procedural costs for redo-SMVR	22,432 (range: 11,216-44864) Gamma (22,432; SD: 5608)	Atluri et al.^26^
Procedural costs for ViV TMVR	11,459 (range: 5730-22918) Gamma (11,459; SD: 2865)	Baron et al.^27^
Valve cost for ViV TMVR	39,654 (range: 19,827-79308) Gamma (39,654; SD: 9914)	Baron et al.^28^
Cost of pacemaker placement	20,507 (range: 10,254-41014) Gamma (20,507; SD: 5127)	Arnold et al.^29^
Cost of arrhythmia	34,623 (range: 17,312-69246) Gamma (34,623; SD: 8656)	Arnold et al.^29^
Cost of bleeding	56,970 (range: 28,485-113940) Gamma (56,970; SD: 14,243)	Arnold et al.^29^
Cost of stroke	40,757 (range: 20,379-81514) Gamma (40,757; SD: 10,189)	Arnold et al.^29^
Cost of renal insufficiency	28,174 (range: 14,087-56348) Gamma (28,174; SD: 7044)	Arnold et al.^29^
Cost of hospitalization per day for the state of Pennsylvania	3279 (range: 1640-6558) Gamma (3279; SD: 820)	Foundation^30^
Cost of lost productivity per day	164 (range: 82 -328) Gamma (164; SD: 41)	Bureau of Labor Statistics^25^
Follow-up costs for ViV TMVR	1178 (range: 589-2356) Gamma (1178; SD: 295)	Galper et al.^31^
Follow-up costs for redo-SMVR	5074 (range: 2537-10148) Gamma (5074; SD: 1269)	Galper et al.^31^
Length of stay (d)		
Total hospital length of stay with ViV TMVR	7.6 (range: 3.8-15.2) Gamma (7.6; SD: 1.9)	Kamioka et al.,^15^ Murzi et al.,^20^ Simonettoet et al.,^16^ Zubarevich et al.^17^
Total hospital length of stay with redo-SMVR	11.6 (range: 5.8-23.2) Gamma (11.6; SD: 2.9)	Kamioka et al.,^15^ Murzi et al.,^20^ Simonettoet et al.,^16^ Zubarevich et al.^17^

Abbreviations: redo-SMVR, redo surgical mitral valve replacement; SD, standard deviation; ViV TMVR, valve-in-valve transcatheter mitral valve replacement.

Probabilities were modeled as beta distributions, whereas costs and LOS were modeled as gamma distributions as recommended by guidelines.^7^ When a range was not available for a variable, we approximated it by allowing the variable to vary between 50% and 200% of its base case value,^32^ a deliberately conservative approach. If a standard deviation (SD) was not available, it was estimated by dividing the range by 6, as suggested for data that do not follow the normal distribution (approximation obtained with the use of Chebyshev inequality).^33^

In the Monte Carlo analysis, which is a simulation method used to quantify uncertainty in model results by repeatedly sampling from probability distributions assigned to uncertain inputs, the model was run 10,000 times^32^ and each time a value from the predetermined distributions (Table 2) was randomly selected for each variable. The results of each simulation were plotted on an incremental cost-effectiveness plane as points with coordinates (x,y), with x representing incremental effectiveness and y representing the incremental cost of ViV TMVR vs. redo-SMVR. Points located within the south-east quadrant of the graph were considered to be dominant.^34^ Dominance in cost-effectiveness analysis occurs when one intervention is both more effective and less costly than the comparator intervention. Finally, cost-effectiveness acceptability curves were generated to illustrate the proportion of probabilistic sensitivity analysis runs in which ViV TMVR was considered the preferable strategy over a range of WTP thresholds.^7^ We used a range of thresholds ranging from $0 to $100,000 in line with commonly accepted thresholds.^35^

## Results

### Base Case Analysis

In the base case analysis, the total cost for the ViV-TMVR strategy was calculated to be $87,724. The overall total effectiveness, measured as the probability of survival with ViV TMVR at 1 month, was estimated at 0.93. Similarly, for the redo-SMVR strategy, the base-case total cost was calculated to be $104,444, and the base-case effectiveness, measured as the probability of survival at 1 month, was 0.89.

The incremental difference in effectiveness between ViV TMVR and redo-SMVR was 0.04 (0.93 *vs*. 0.89). Overall, ViV TMVR was associated with one fewer death per 25 patients treated compared to redo-SMVR. ViV TMVR was also associated with lower costs, resulting in an incremental cost difference of −$16,720 ($87,724 vs. $104,444). ViV TMVR resulted in savings of $418,001 per death averted (ICER, −$418,001/death averted) compared to redo-SMVR, suggesting that it is a cost-effective strategy. Together, these findings yielded a negative ICER of −$418,001 per death averted, indicating that ViV TMVR is both more effective and less costly than redo-SMVR and therefore represents a dominant strategy at the modeled time horizon.

### Sensitivity Analysis

**Figure 2:**
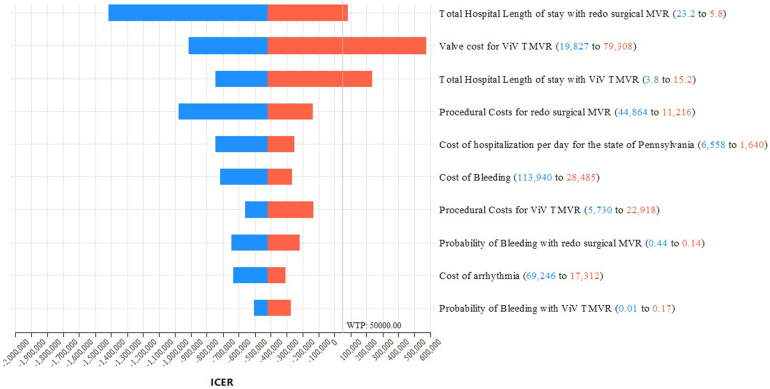
**Tornado diagram.** This graph is a summary of the one-way sensitivity analysis evaluating the impact of individual model parameters on ICER for ViV TMVR vs. redo-SMVR. Each horizontal bar represents the range of ICER values obtained when a single parameter is varied across its predefined lower and upper bounds while all other parameters are held constant at their base-case values. From top to bottom, it presents the variables that led to the greatest change in the ICERs. Blue bars correspond to the ICER calculated at the lower bound of the parameter range, and red bars correspond to the ICER at the upper bound. The numeric ranges shown in parentheses next to each parameter indicate the lower and upper values used in the sensitivity analysis. The vertical reference line labeled at a willingness-to-pay (WTP) threshold of $50,000 represents the commonly cited cost-effectiveness threshold; ICER values to the left of this line are considered cost effective at this threshold, whereas values to the right exceed it. Abbreviations: ICER, incremental cost-effectiveness ratio; MVR, mitral valve replacement; redo-SMVR, redo surgical mitral valve replacement; ViV TMVR, valve-in-valve transcatheter mitral valve replacement.

The one-way sensitivity analysis, which allowed us to test each model variable for thresholds by varying each base-case value within the limits specified in Table 2, suggested that in clinical practice compared to ViV TMVR, the redo-SMVR strategy would become the cost-effective option if the valve cost is higher than $56,374, the LOS for redo-SMVR is less than 6.7 days, the LOS for ViV TMVR is greater than 12.5 days, or the mortality probability with ViV TMVR is greater than 0.11. The findings of the sensitivity analysis were summarized in the tornado diagram (Figure 2), which is a graphical representation of how variations in each model variable affect the cost-effectiveness output. More specifically, our one-way sensitivity analyses demonstrated that cost-effectiveness was driven primarily by differences in resource utilization rather than uncertainty in effectiveness. As shown in the tornado diagram, the most influential parameters were hospital LOS for redo-SMVR, intensive care unit LOS, and total procedural costs followed by the device cost of the ViV TMVR valve. Variation in these inputs produced the greatest changes in the ICER.

Moreover, we estimated the ICER for various mortality probabilities of ViV TMVR. These were ICER −$196,706 per death averted at a mortality of 2.5%, ICER −$278,667 per death averted at a mortality of 5%, and ICER −$1,672,004 per death averted at a mortality of 10%. In addition, as ViV TMVR valve cost varies significantly among institutions, we also included a graph depicting the relationship between valve cost and ICER (Supplementary Figure S2). Furthermore, we varied the mortality probability estimates outside the ranges specified in Table 2. Supplementary Figure S3 shows the ICERs for various redo-SMVR probabilities of mortality (between 0 and 0.20) whereas Supplementary Figure S4 showed the ICERs for various ViV TMVR mortality probabilities.

### Probabilistic Analysis

**Figure 3:**
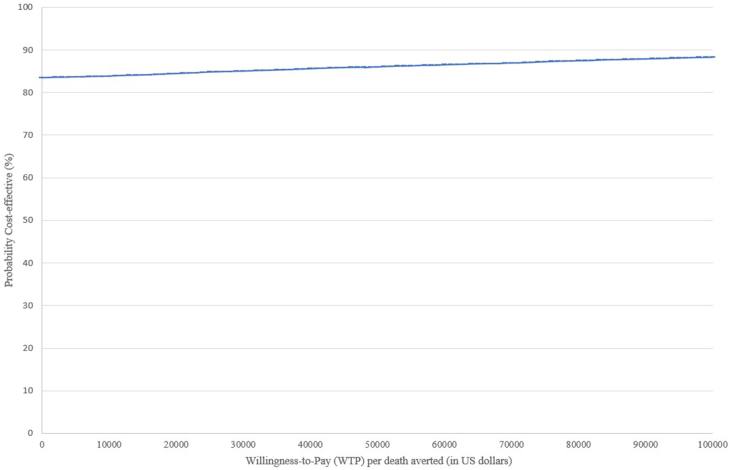
**Cost-effectiveness acceptability curve with a willingness to pay ranging from $0 to $100,000.** This curve shows the probability that ViV TMVR is a cost-effective strategy compared to redo-SMVR, the baseline strategy, for a range of different cost-effectiveness thresholds. Abbreviations: redo-SMVR, redo surgical mitral valve replacement; ViV TMVR, valve-in-valve transcatheter mitral valve replacement.

In the probabilistic analysis, the mean cost for ViV TMVR was estimated to be $87,569 (95% prediction interval $87,295-$87,843) and the mean cost for redo-SMVR was estimated to be $104,593 (95% prediction interval $104,273-$104,912). In the cost-effectiveness acceptability curve (Figure 3), we show the probability that ViV TMVR is cost-effective compared to redo-SMVR for various WTP thresholds. ViV TMVR was cost-effective in 83% to 88% of simulations for a WTP ranging from $0 to $100,000.

## Discussion

In this analysis, we found that ViV TMVR is a cost-effective strategy for the management of degenerated bioprosthetic MVs given that it resulted in savings of $418,001 per death averted (ICER, −$418,001/death averted) compared to redo-SMVR. Furthermore, ViV TMVR remained cost effective across multiple sensitivity analyses and in 83% to 88% of simulations for a WTP ranging from $0 to $100,000.

Given that ViV TMVR is an emerging intervention, its financial implications have not yet been systemically analyzed through a cost-effectiveness analysis for a US-based medical setting. A prior study comparing the two strategies using the National Readmission Database had estimated the median cost of hospitalization at $60,670 for ViV TMVR and $67,232 for redo-SMVR.^23^ A Canadian-based health technology assessment analysis published by Ontario^36^ Health estimated that publicly funding transcatheter valve-in-valve implantation for failing mitral or tricuspid bioprosthetic valves could lead to cost savings of $0.33 million over 5 years. In this study, ViV TMVR was associated with in-hospital, 30-day, and 1-year mortality rates of 0% to 5%, 0% to 15%, and 14% to 27%, respectively. Notably, the prior analyses primarily describe resource utilization or aggregate budget impact and do not integrate costs with clinical effectiveness to assess value. Our study aligns with prior reports of favorable short-term outcomes after ViV TMVR and extends the cost analysis to a US health care system. It adds to the existing literature by combining US-specific cost data with short-term mortality outcomes in a transparent decision-analytic model, allowing estimation of incremental cost-effectiveness rather than cost or outcomes alone.

The cost-effectiveness of ViV TMVR can be attributed to several factors. Firstly, it is minimally invasive compared to redo-SMVR^15^, ^16^, ^17^^,^^20^ and thereby allows for an earlier hospital discharge and recovery. Secondly, it is associated with fewer periprocedural complications including stroke, bleeding, acute kidney injury, arrhythmias, and permanent pacemaker insertion.^4^ Together, these factors contribute to reduced overall costs by decreasing hospital stays, lowering the risk of complications, and improving the rapidity of patient recovery and quality of life.

Our sensitivity analysis underscored the model's resilience and dependability across a wide array of ranges and distributions. Notably, ViV TMVR remains cost effective for mortality of up to 11%. Furthermore, the tornado diagram provided a graphical depiction of uncertainty, aiding in the identification of pivotal drivers influencing the cost-effectiveness outcomes. Specifically, the most influential variables were TMVR valve cost, procedural costs for redo-SMVR, and hospitalization expenses per day.

Although our study offers valuable insights, several limitations should be considered. Firstly, cost-effectiveness analyses depend on mathematical models, which inherently come with modeling and statistical limitations. Moreover, the effectiveness data incorporated in this study were obtained from retrospective, observational studies, which are subject to residual confounding and selection bias unlike randomized controlled trials. As a case in point, the mortality estimates we have used, particularly for the surgical arm, are slightly higher than others reported in the literature that may not fully represent real-world clinical practice. Quality-adjusted life years (QALYs) were not included in our analysis, as there are no reliable estimates in the present literature. In addition, precise cost data for ViV TMVR were unavailable, as these figures are not yet published in the existing literature. For similar reasons, we have not distinguished with respect to the type of prior implant, such as for different ring designs, and we have not accounted for access route such as transapical, transeptal, or both. Furthermore, we did not account for intensive care unit LOS and its associated costs due to the absence of this data in the literature. Including these costs could potentially highlight further savings for ViV TMVR, underscoring an area for future investigation. Similarly, the costs assigned for redo-SMVR were based on those for the first-time MVR surgeries. Since redo-SMVR is typically associated with higher costs and longer LOSs compared to the first-time SMVR, the ViV TMVR strategy may offer even greater potential savings. Notably, our analysis only applies to patients who are candidates for both ViV TMVR and redo-SMVR and does not account for specific subgroups of the eligible population. However, the cost data we have used may not be reflective of a population that is eligible for both procedures. Our analysis also used a short time horizon, as it provided us with the most robust and reliable data. However, because most costs and complications are seen periprocedurally, the long-term impact on costs may be minimal. However, some evidence suggests that the immediate benefits of ViV TMVR may be eventually offset by valve events and long-term survival relative to redo-SMVR^37^; this currently renders the long-term cost-effectiveness unclear. In addition, although we have accounted for lost productivity costs, notably, we have not included losses associated with uncompensated household production. Incorporating these factors could potentially enhance the costeffectiveness of the ViV TMVR strategy. Similarly, in our lost productivity estimations, we have only accounted for the days patients are hospitalized. We have not accounted for specific information such as demographics, functional recovery, and return-to-work timing. This approach likely underestimates the true productivity costs associated with redo-SMVR, as these patients typically experience a longer recovery period and extended time off work. To address all the above limitations, we have accounted for potential variations in base-case model inputs with sensitivity and probabilistic analyses, both of which underscored the model's resilience and reliability.

As the prevalence of bioprosthetic valves is expected to continue to rise, identifying cost-effective strategies for the management of valve degeneration is of particular importance. Our study, which integrates effectiveness and cost data from the literature, establishes that ViV TMVR is a cost-effective option, with shorter hospital stays being the primary drivers of the finding. With the growing use of transthoracic echocardiography and intracardiac echocardiography to guide the ViV TMVR procedure as alternatives to transesophageal echocardiography under general anesthesia, there may be opportunities to reduce anesthesia-related costs and resource utilization.^38^ However, the net cost impact remains uncertain given the added device cost of intracardiac echocardiography catheters^39^ and variability in procedural practices. Future cost-effectiveness studies should aim to incorporate information about valve durability, repeat interventions, long-term survival, and quality-of-life measures and report cost-utility outcomes (e.g., cost per QALY gained). Moreover, supported by data from the Valve-in-Valve registry, ViV TMVR is now approved for both high- and intermediate-risk patients.^40^ Ongoing and future registry-based studies should capture granular procedural data, comprehensive cost breakdowns, and long-term clinical outcomes for both ViV TMVR and redo-SMVR to support more refined, patient-level economic evaluations as this technology continues to evolve.

## CRediT Authorship Contributions

All authors approved and contributed to this manuscript.

## Ethics Statement

This study did not require IRB approval as all included data was obtained from the published literature.

## Funding

The authors have no funding to report.

## Disclosure Statement

Paul Fiorilli has received consulting honoraria from 10.13039/100006520Edwards Lifesciences. Suzanne J. Baron received research support from 10.13039/100020297Abiomed and 10.13039/100008497BSC; served on advisory boards for 10.13039/100015345Zoll Medical, 10.13039/100004374Medtronic, 10.13039/100008497BSC, 10.13039/100006520Edwards Lifesciences, and 10.13039/100020297Abiomed; and received speaking honoraria from Shockwave, 10.13039/100015345Zoll Medical, 10.13039/100004374Medtronic, and 10.13039/100008497BSC. Jay Giri has received research funding and served on advisory boards for 10.13039/100020297Abiomed, 10.13039/100006520Edwards, 10.13039/100008497Boston Scientific, and 10.13039/100022880Inari Medical; and holds equity in Endovascular Engineering. Ashwin S. Nathan has received research funding from 10.13039/100020297Abiomed, Biosense, Webseter, 10.13039/100006520Edwards Lifesciences, and 10.13039/100008497Boston Scientific; and he has received speaking fees from 10.13039/100006520Edwards Lifesciences.

The other authors had no conflicts to declare.
