# Supplementary material for: Cost-Effectiveness of Valve-in-Valve Transcatheter Mitral Valve Replacement Versus Redo Surgical Mitral Valve Replacement for Degenerated Bioprosthetic Mitral Valve
Source: Struct Heart. 2026 Feb 9;10(4):100808. doi: 10.1016/j.shj.2026.100808 (PMC12997312; doi:10.1016/j.shj.2026.100808)
Supplement: Supplementary Materials [file mmc1.docx]

**Supplemental Materials**


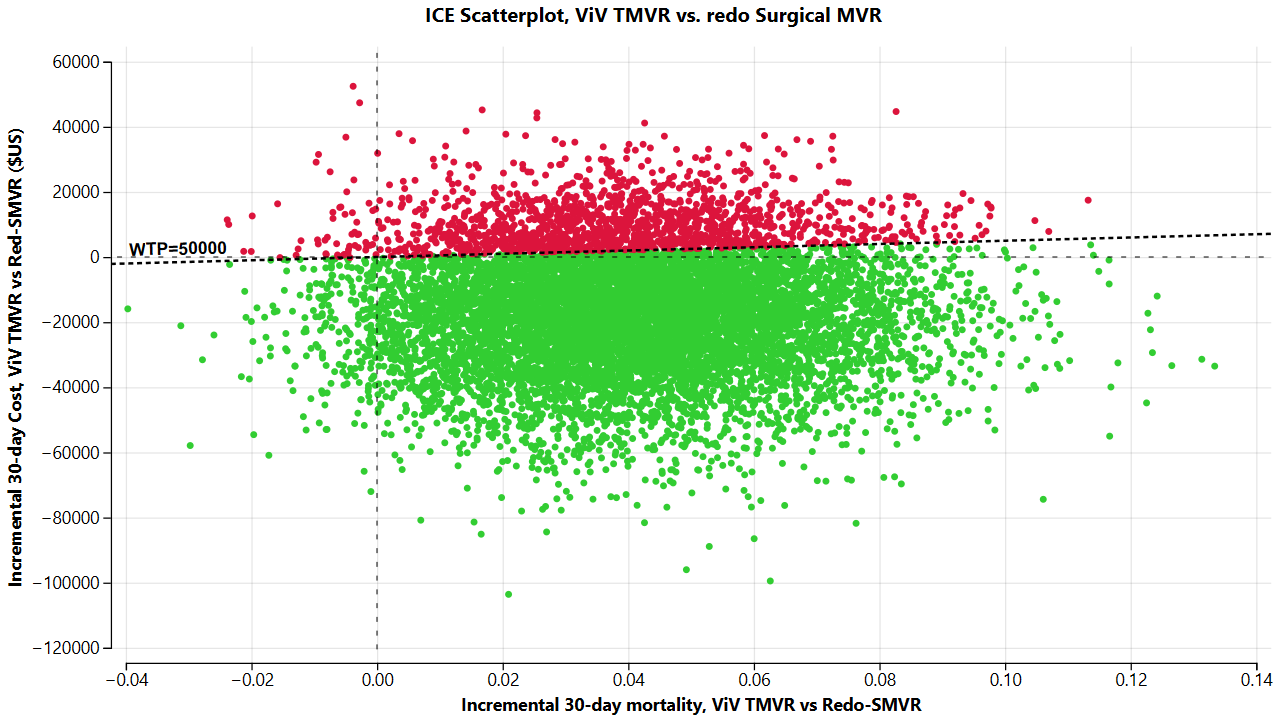


**Figure S1.** Incremental cost-effectiveness acceptability plane. This figure displays results from the probabilistic sensitivity analysis (PSA) of the decision-analytic model comparing valve-in-valve transcatheter mitral valve replacement (ViV TMVR) with redo surgical mitral valve replacement (redo-SMVR). Each point represents the incremental cost and incremental effectiveness from a single Monte Carlo simulation (10,000 iterations), with values generated by randomly sampling from predefined probability distributions for all model parameters. The x-axis represents incremental effectiveness (difference in mortality between ViV TMVR and redo-SMVR), with positive values indicating improved survival for ViV TMVR. The y-axis represents incremental cost (difference in total cost), with negative values indicating cost savings for ViV TMVR. Points in the southeast quadrant represent simulations in which ViV TMVR is both more effective and less costly (dominant) compared with redo-SMVR. Points in the northeast quadrant represent simulations in which ViV TMVR is more effective but more costly. The vertical dashed line at zero denotes no difference in effectiveness, and the horizontal dashed line at zero denotes no difference in cost. The diagonal dashed line represents a willingness-to-pay (WTP) threshold of $50,000 per unit of effectiveness gained; points below this line are considered cost effective at this threshold. The ellipse illustrates the joint distribution of incremental costs and effects, capturing the majority of PSA iterations and highlighting the concentration of simulations favoring ViV TMVR as a cost-saving strategy.

**
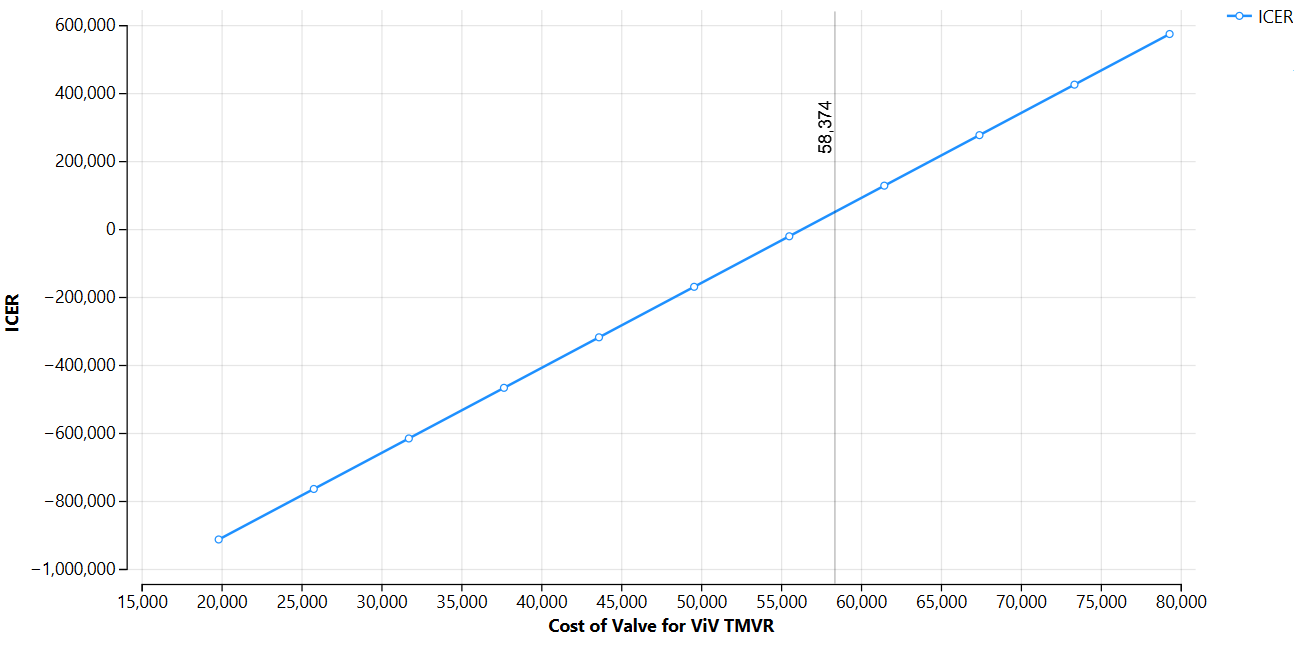
**

**Figure S2**. The relationship between valve-in-valve transcatheter mitral valve replacement (ViV TMVR) valve cost and incremental cost-effectiveness ratio (ICER). This figure illustrates a one-way sensitivity analysis examining the impact of variation in the valve cost for ViV TMVR on the ICER compared with redo surgical mitral valve replacement (redo-SMVR). The x-axis represents the assumed unit cost of the ViV TMVR valve, varied across the plausible range used in the model. The y-axis represents the resulting ICER, expressed as incremental cost per unit of effectiveness gained.


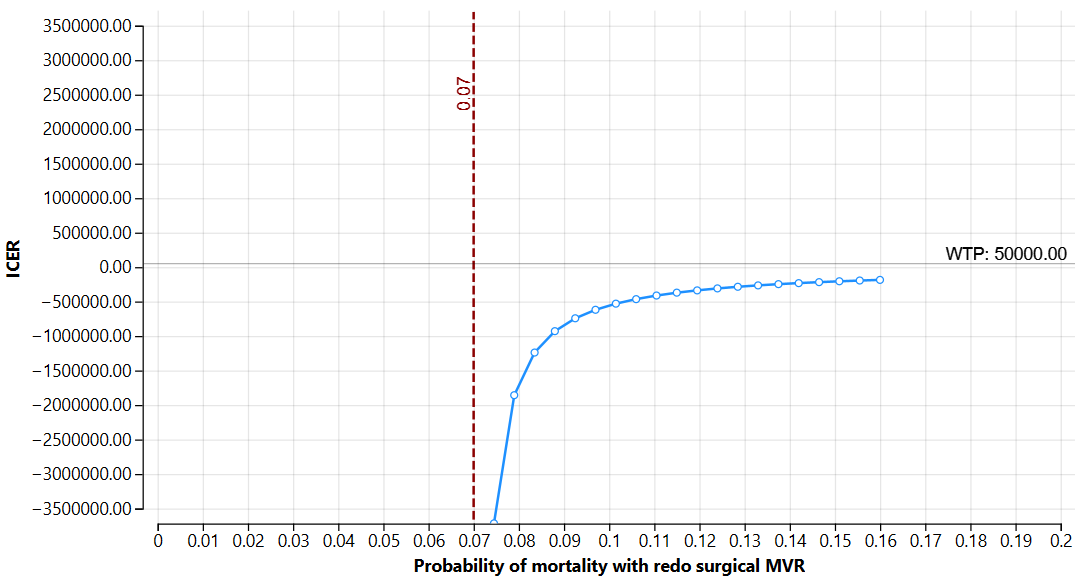
**Figure S3**. Incremental cost-effectiveness ratios (ICERs) (y-axis) for various probabilities of mortality with redo surgical mitral valve replacement (redo-SMVR) (x-axis).


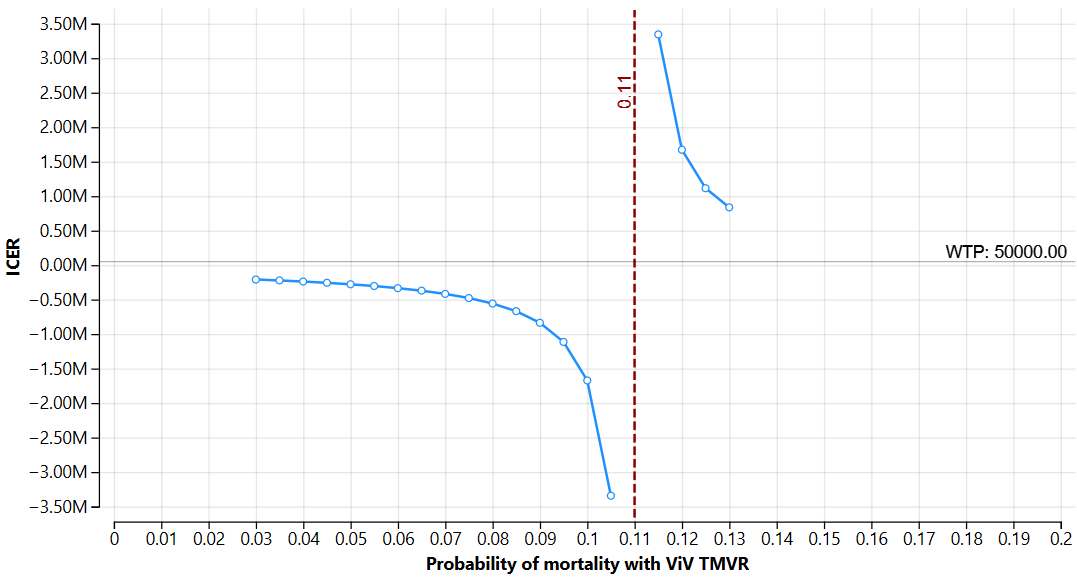
**Figure S4**: ICERs (y-axis) for various probabilities of mortality with ViV TMVR (x-axis).
